# Trust – IoV: An open benchmark dataset for trust management in the internet of vehicles

**DOI:** 10.1016/j.dib.2026.112578

**Published:** 2026-02-13

**Authors:** Yingxun Wang, Adnan Mahmood, Xiang Wang

**Affiliations:** a Faculty of Computer and Information Engineering, Qilu Institute of Technology, Jinan, 250200, Shandong, PR China; b School of Computing, Macquarie University, Sydney, NSW 2109, Australia

**Keywords:** Internet of Vehicles, V2X communication, Network security, Trust management, Trust parameters, Trust-based attacks

## Abstract

Whilst advancements in information and communication technologies and artificial intelligence have considerably enhanced the capabilities of the Internet of Vehicles (IoV) paradigm, ensuring the security of this highly dynamic and decentralized network still remains a critical challenge. Accordingly, the notion of trust has been lately employed by researchers in academia and industry with an intent to primarily tackle the internal attacks that have the potential to considerably jeopardize the security of an IoV network. However, as of date, there is a lack of publicly released trust-based IoV datasets particularly designed for detecting trust-based attacks. Therefore, this paper envisages a novel trust-based IoV dataset encompassing 96 vehicles, i.e., both trustworthy and intelligent malicious, with the latter one equipped with the ability to instigate trust-based attacks (on-off attacks, self-promoting attacks, and opportunistic attacks). A total of 1,048,576 interactions have been carried out by the said vehicles at different time instances. Four salient trust parameters, i.e., experience of interaction, frequency of interaction, timeliness of interaction, and quality of received messages, have been employed to quantify trust of each interaction segment of the vehicles. As the first trust-based IoV dataset encompassing information pertinent to the trust-based attacks, it provides a robust basis for advancing future research in this specialized domain.

Specifications Table

**Table:** 

Subject	Computer Sciences
Specific Subject Area	Internet of Vehicles, Trust Management
Type of Data	Tabular
Data Collection	This dataset has been simulated via a Java-based IoV simulator that employed a realistic urban traffic scenario, i.e., of Shenzhen, Guangdong Province, P. R. China. A total of 96 vehicles, encompassing trustworthy and intelligent malicious ones, traverse along various road segments at random speeds. Vehicles interact with other vehicles in their immediate neighborhood and each interaction has been quantified via four salient trust parameters, i.e., experience of interaction, frequency of interaction, timeliness of interaction, and quality of received messages. In total, 1,048,576 interactions have been recorded amongst the vehicles. Intelligent malicious vehicles have been programmed to carry out trust-based attacks, i.e., on-off attacks, self-promoting attacks, and opportunistic attacks, to simulate real-world trust-based malicious behaviors.
Data Source Location	Qilu Institute of Technology, Jinan, 250200, Shandong, P. R. China
Data Accessibility	The preprocessed dataset is publicly available for reproducibility. Repository Name: Zenodo Data identification number: 10.5281/zenodo.17636200 Direct dataset link: https://doi.org/10.5281/zenodo.17636200

## 1 Value of the Data

• As of date, there remains a considerable lack of publicly accessible trust-based IoV datasets that could be utilized by the research community. This scarcity has constituted a critical bottleneck and persistently impedes the evaluation of trust-based mechanisms in the context of an IoV network.
• This particular trust-based IoV dataset is well-suited for assessing the trustworthiness of vehicles. By weighted aggregation of the trust parameters, i.e., experience of interaction, frequency of interaction, timeliness of interaction, and quality of received messages, a total trust score can be ascertained for each vehicle at each time instance. If the total trust score for a particular vehicle exceeds the stipulated trust threshold, it is classified as trustworthy or untrustworthy otherwise. Trust parameters can also be analyzed via intelligent learning-based algorithms and the classification pertinent to trustworthy and untrustworthy vehicles can be effectively distinguished via an optimal decision boundary.
• This dataset encompasses the simulation of various trust-based attacks, i.e., on-off attacks, self-promoting attacks, and opportunistic attacks, thereby assisting researchers from both academia and industry to validate the robustness of their respective IoV-based trust mechanisms. In case of an on-off attack, a malicious vehicle switches between trustworthy and untrustworthy behavior so as to evade detection. Similarly, a self-promoting attack is employed by a malicious vehicle via falsified positive feedback from the sybil identities to artificially inflate its respective reputation with the key intent being to gain more privileges in an IoV network. Moreover, an opportunistic attack is launched by an intelligent malicious vehicle for abruptly taking down the applications and services of the vehicles relying on it so as to inflict maximum harm on the said vehicles, and that too, within a shortest possible duration to avoid detection.
• This particular IoV dataset has a potential to be employed for trustworthy participant selection in the context of federated learning frameworks. It is pertinent to highlight that only reliable participants can ensure the integrity and reliability of this collaborative learning approach, and reduce the risks associated with the data poisoning attacks.

## 2 Background

IoV manifests a highly dynamic communication network that facilitates interactions between vehicles via vehicle-to-vehicle communication, vehicles and supporting roadside infrastructure via vehicle-to-infrastructure communication, vehicles and vulnerable pedestrians via vehicle-to-pedestrian communication, and vehicles and backbone network via vehicle-to-network communication, thereby realizing Vehicle-to-Everything (V2X) communication [1,2]. The motivation for designing this trust-based IoV dataset arises from the growing demand for secure communication in an IoV network. As vehicles increasingly depend on V2X communication, trust management becomes highly indispensable for preventing malicious behaviors to ensure traffic safety [[3], [4], [5]]. Owing to the inherent dynamic characteristic of an IoV network, conventional security mechanisms often fail to address the unique requirements of the said network [6,7]. This IoV dataset has been, therefore, envisaged for assessing the trustworthiness of dynamic interactions between vehicles. Accordingly, a realistic driving scenario under various dynamic traffic conditions has been employed and by particularly incorporating both trustworthy and malicious behaviors. This dataset encompasses four salient trust parameters, i.e., experience of interaction, frequency of interaction, timeliness of interaction, and quality of received messages, to support both conventional and a learning-based approach, thereby offering researchers a standardized benchmark for evaluating their respective trust models for improving the resilience of an IoV network.

## 3 Data Description

Over the past decade or so, the notion of trust has become a focal point for researchers across academia and industry [8,9]. Trust, in essence, refers to the probability of a trustee to realize a certain action or a set of services, i.e., requested by a trustor, in an appropriate manner at a particular instance of time [10]. Trust values, expressed within the range of [0, 1], serve as a quantitative measure for evaluating the trustworthiness of a trustee with 0 implying untrustworthiness and 1 suggesting trustworthiness [11]. Accordingly, the manuscript-at-hand presents an IoV simulator envisaged via Java and which has been made publicly accessible to readers.^1^ To ensure a realistic outlook, we have not only incorporated trustworthy vehicles but have also catered for malicious vehicles which dynamically switch between trustworthy and untrustworthy behaviors to execute trust-based attacks in order to evade detection in the context of an IoV network.

The envisaged trust-based IoV dataset encompasses 96 vehicles that have carried out 1,048,576 interactions in total, i.e., both positive and negative, at different time instances. The envisaged dataset is in a tabular format and comprises 10 columns, i.e., Interaction Time, Trustor, Trustee, four trust parameters (Experience of Interaction, Frequency of Interaction, Timeliness of Interaction, and Quality of Received Messages), Total Trust, and two labels (MaliciousVehicle and IsAttacking). The details are presented as follows:

### 3.1 Trustor

The trust between vehicles is ascertained by integrating multiple trust parameters which are primarily quantified based on the relationship between them. Accordingly, the vehicle that intends to act as an evaluator so as to assess and determine the trustworthiness of a target vehicle is defined as a trustor. The same is depicted in the second column of our envisaged trust-based IoV dataset.

### 3.2 Trustee

The target vehicle, i.e., the one whose trustworthiness is being ascertained, is referred as a trustee. In our envisaged trust-based IoV dataset, trustees have been delineated in the third column. It is pertinent to mention that 94 trustees have engaged in a total of 1,048,576 interactions with trustors in the context of the said dataset.

### 3.3 Experience of interaction (IExp)

Experience of Interaction (0 ≤ IExp ≤ 1) manifests the interaction-based history between a trustor and a trustee in an IoV network, and is, therefore, measured at a time instance *t* by taking into account the proportion of the total number of positive interactions between a trustor and a trustee vis-à-vis the total number of interactions amongst them up to that particular time. IExp is presented in the column four of our envisaged trust-based IoV dataset.

### 3.4 Frequency of interaction (IFre)

Frequency of Interaction (0 ≤ IFre ≤ 1) suggests how often a trustor interacts with a trustee at a time instance *t*, and is quantified by considering the proportion of the total interactions between a trustor and a trustee to the trustor’s total interactions with all other trustees up to that particular time. A higher frequency of interactions thus contributes to more accurately determining the true characteristics of a trustee. IFre is depicted in the column five of our envisaged trust-based IoV dataset.

### 3.5 Timeliness of interaction (ITim)

Timeliness of Interaction (0 ≤ ITim ≤ 1) signifies the time of interaction between a trustor and a trustee with respect to the current time. Due to the inherent highly dynamic characteristics of an IoV network, prioritizing recent interactions over previous ones is crucial. Accordingly, in the design of this IoV simulator, considerable attention was accorded to this particular parameter. ITim is illustrated in the column six of our envisaged trust-based IoV dataset.

### 3.6 Quality of received messages (RMQ)

Quality of Received Messages (0 ≤ RMQ ≤ 1) primarily depends on the network communication quality within an IoV network and, therefore, has a direct impact on the quality of messages that a trustee receives from a trustor at a time instance *t*. A higher network communication quality typically suggests that a trustor can ascertain the trustworthiness of a trustee in a more precise manner. RMQ is outlined in the column seven of our envisaged trust-based IoV dataset.

### 3.7 Total trust

The total trust of a particular vehicle is quantified via the above proposed four trust parameters, i.e., experience of interaction, frequency of interaction, timeliness of interaction, and quality of received messages. In our envisaged trust-based IoV dataset, a weighted sum approach has been employed to aggregate the said trust parameters. When a vehicle’s total trust exceeds the stipulated threshold, the said vehicle is considered trustworthy, otherwise, it is deemed untrustworthy. Furthermore, the total trust value can also be used to evaluate a vehicle’s behavior over time, i.e., by detecting temporal changes in a vehicle’s behavior, different types of trust-based attacks instigated by the said vehicle, if malicious, can be identified. This, therefore, enables the detection and subsequent elimination of malicious vehicles from an IoV network. In the envisaged trust-based IoV dataset, the total trust is depicted in column eight.

### 3.8 MaliciousVehicle

MaliciousVehicle is a label employed to indicate the behavior of a vehicle within our envisaged trust-based IoV dataset, i.e., ‘Yes’ denotes an attacking vehicle, whereas, ‘No’ indicates a normal, non-malicious vehicle. MaliciousVehicle is presented in the column nine of our envisaged trust-based IoV dataset.

### 3.9 IsAttacking

IsAttacking is a label that illustrates whether a vehicle is currently in an attacking state. ‘Yes’ implies that a vehicle is actively performing a trust-based attack, whereas, ‘No’ suggests that a vehicle is playing intelligently by engaging in a normal interaction. IsAttacking is delineated in the column ten of our envisaged trust-based IoV dataset.

## 4 Experimental Design, Materials and Methods

**Fig. 1:**
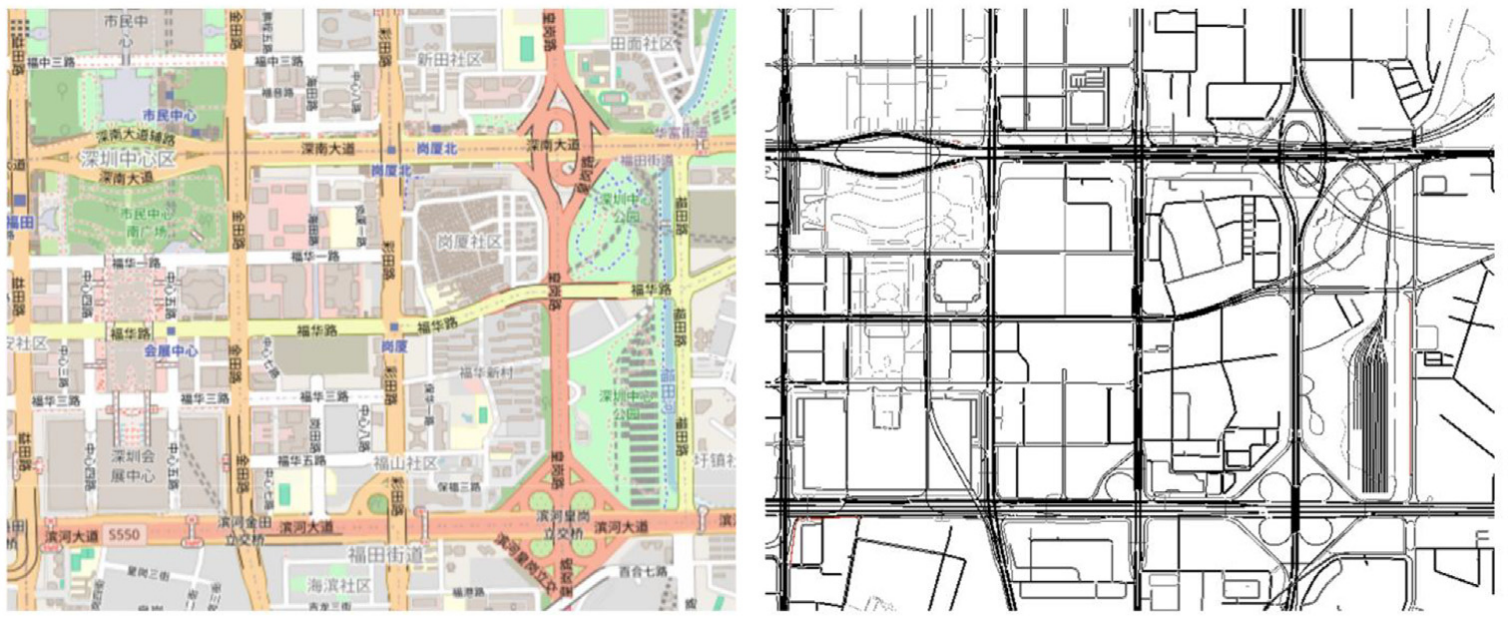
A realistic urban scenario of Shenzhen, Guangdong Province, P.R. China.

It is pertinent to highlight that, as of date, no publicly accessible trust-based IoV dataset has been particularly designed for detecting trust-based attacks. Therefore, the dataset envisaged in this manuscript makes a pioneering contribution to this specific domain. For the purpose of generating the said dataset, Java has been utilized to develop an IoV simulator which employed a realistic urban traffic scenario encompassing multiple interconnected road segments, i.e., via a hybrid structure of grid style main roads (6 major intersections) and complex multi-level interchanges (2 overpasses), of Shenzhen, Guangdong Province, P. R. China (Fig. 1). A total of 96 vehicles, i.e., trustworthy and intelligent malicious, have been instantiated and subsequently facilitated for interaction in the said traffic scenario, wherein vehicles move at random speeds along different trajectories. Each vehicle maintains a constant speed along a particular trajectory and changes its respective speed on switching to a new trajectory [12]. In doing so, they interact with other vehicles in their immediate neighborhood. Accordingly, each interaction is quantified via four salient trust parameters, i.e., experience of interaction, frequency of interaction, timeliness of interaction, and quality of received messages, at each time instance. Tables 1 and Table 2 present a specimen of our envisaged trust-based IoV dataset to facilitate readers to understand the same. Specifically, the intelligent malicious vehicles are programmed to carry out trust-based attacks – on-off attacks (an intelligent malicious vehicle alternates between honest and dishonest modes), self-promoting attacks (an intelligent malicious vehicle colludes to enhance its respective reputation for gaining significant privileges), and opportunistic attacks (an intelligent malicious vehicle gains considerable trust of vehicles interacting with it and subsequently finds an opportunity to harm them).

**Table 1:** A specimen of the envisaged trust-based IoV dataset encompassing the trust parameters, i.e., IExp – Experience of Interaction, IFre – Frequency of Interaction, ITim – Timeliness of Interaction, and RMQ – Quality of Received Messages, and labels, i.e., MaliciousVehicle and IsAttacking.

Trustor	Trustee	IExp	IFre	ITim	RMQ	MaliciousVehicle	IsAttacking
0	8	1.000	0.500	1.000	1.000	No	No
	24	0.400	0.143	1.000	0.600		
	92	0.800	0.071	1.000	0.800		
.	.	.	.	.	.	.	.
10	15	0.900	0.111	1.000	1.000	No	No
	57	0.300	0.001	0.244	0.600		
	81	0.600	0.100	1.000	0.600		
.	.	.	.	.	.	.	.
19	20	0.300	0.013	0.653	0.600	No	No
	25	0.300	0.006	0.326	0.600		
	92	0.800	0.125	1.000	0.800		
.	.	.	.	.	.	.	.
36	39	0.500	0.012	1.000	0.600	No	No
	61	0.300	0.001	0.249	0.600		
	89	0.400	0.002	0.498	0.600		
.	.	.	.	.	.	.	.
42	58	0.700	0.111	1.000	0.800	No	No
	75	0.600	0.022	1.000	0.600		
	90	0.400	0.007	0.965	0.600		
.	.	.	.	.	.	.	.
61	63	1.000	0.048	1.000	1.000	No	No
	82	0.400	0.005	0.684	0.600		
	93	0.700	0.097	1.000	0.800		
.	.	.	.	.	.	.	.
73	75	0.900	0.098	1.000	1.000	No	No
	76	0.800	0.267	1.000	0.800		
	87	1.000	0.048	1.000	1.000		
.	.	.	.	.	.	.	.
80	81	0.400	0.012	0.895	0.600	Yes (*on-off attack)*	Yes
	88	0.900	0.226	1.000	1.000		
	93	0.800	0.173	1.000	0.800		
.	.	.	.	.	.	.	.
85	87	0.300	1.000	0.392	0.600	Yes (*self-promoting attack)*	Yes
	92	0.700	0.268	0.941	0.800		
	95	0.700	0.031	0.521	0.800		
.	.	.	.	.	.	.	.
90	91	0.700	0.243	1.000	0.800	Yes (*on-off attack)*	Yes
	93	0.300	0.074	1.000	0.600		
	95	0.600	1.000	1.000	0.600

**Table 2:** The values of the trust parameters, i.e., IExp – Experience of Interaction, IFre – Frequency of Interaction, ITim – Timeliness of Interaction, and RMQ – Quality of Received Messages, and labels, i.e., MaliciousVehicle and IsAttacking, of vehicle 40 and vehicle 91.

Trustor	Trustee	IExp	IFre	ITim	RMQ	MaliciousVehicle	IsAttacking
40	41	0.700	1.000	1.000	0.800	No	–
	44	0.300	0.872	1.000	0.600		
	47	0.800	0.047	1.000	0.800		
	54	0.600	0.006	0.923	0.600		
	56	0.700	0.004	0.955	0.800		
	58	0.700	0.042	1.000	0.800		
	60	0.500	0.004	0.774	0.600		
	63	0.900	0.008	1.000	1.000		
	69	0.600	0.015	1.000	0.600		
	71	0.300	0.006	0.720	0.600		
	75	0.900	0.024	1.000	1.000		
	77	0.500	0.019	1.000	0.600		
	82	0.400	0.004	0.905	0.600		
	85	0.300	0.007	0.745	0.600		
	88	0.500	0.003	0.997	0.600		
	90	1.000	0.020	1.000	1.000		
	92	0.800	0.016	1.000	0.800		
	93	0.900	0.004	0.887	1.000		
91	93	0.600	0.975	1.000	0.600	Yes (*opportunistic attack)*	Yes
	93	0.800	0.975	1.000	0.800		
	93	0.800	0.985	1.000	0.800		
	93	0.800	0.999	1.000	0.800		
	93	0.200	0.050	0.233	0.100		
	93	1.000	0.800	1.000	1.000		
	93	0.800	0.975	1.000	0.800		
	93	1.000	0.975	1.000	0.800		
	93	0.900	0.975	1.000	1.000		
	95	0.900	0.975	1.000	1.000		

**Fig. 2:**
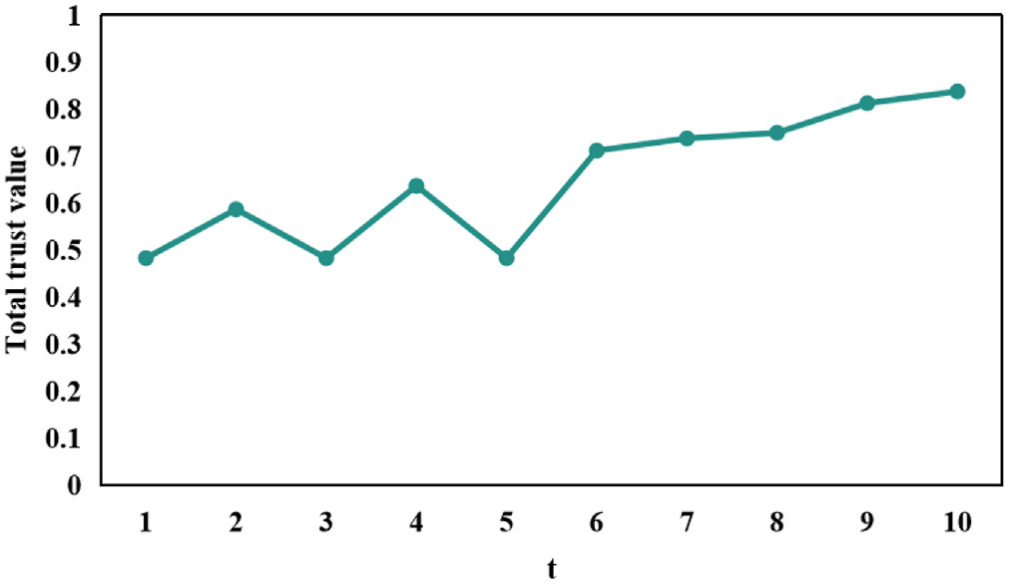
The total trust score of vehicle 84 over each of the 10 time instances.

**Fig. 3:**
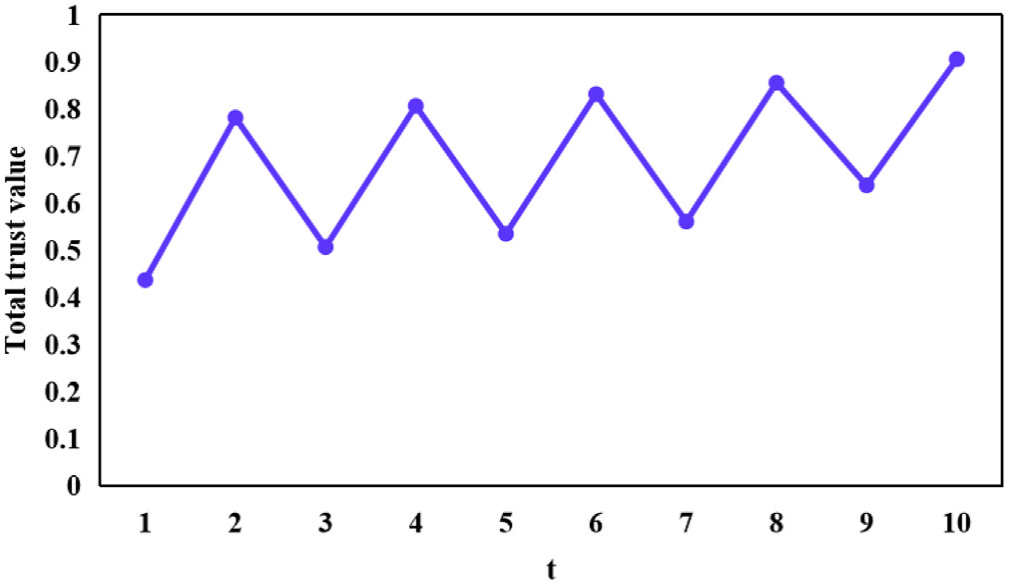
The total trust score of vehicle 87 over each of the 10 time instances.

**Fig. 4:**
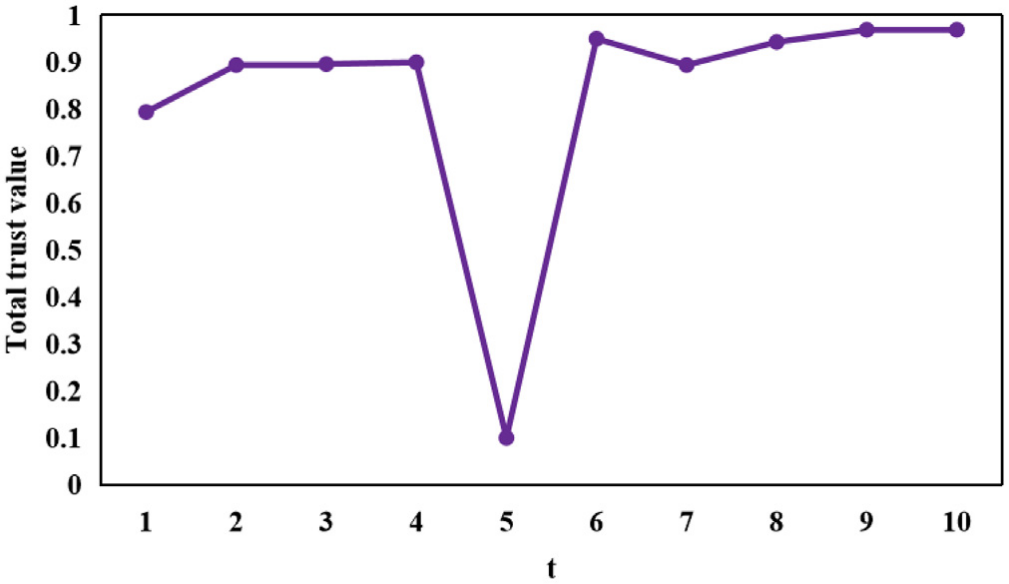
The total trust score of vehicle 91 over each of the 10 time instances.

Fig. 2, Fig. 3, Fig. 4 portray the total trust of vehicle 84, vehicle 87, and vehicle 91, respectively over 10 time instances. It is pertinent to mention here that vehicle 84 instigated an on-off attack along with a self-promoting attack, whereas, vehicle 87 only launched a classical on-off attack. In doing so, both of these vehicles maintained a considerably high trust scores, thereby disguising themselves as trusted entities within an IoV network and consequently disseminating malicious information within the same. This, therefore, can prove extremely fatal in the context of safety-critical applications. Moreover, vehicle 91 instigated a classical opportunistic attack since it possesses a considerable reputation within the context of an IoV network due to its high trust score and is, therefore, an optimal candidate which other vehicles can rely upon in a bid to realize their respective requisite applications and services. The same is manifested at time instance 5 in Fig. 4, wherein vehicle 91 took the opportunity to harm vehicles relying on it.

## Limitations

Owing to the inherent highly dynamic characteristics of an IoV network, designing a trust-based IoV dataset is far from simple. Accordingly, the envisaged trust-based IoV dataset would become an extremely valuable resource for researchers to conduct further studies related to trust management in an IoV network. Below, we outline the main challenges encountered during the dataset creation process:

• Limitations of the trust parameters – Despite selecting a set of salient trust parameters, it remains extremely difficult to fully capture the complexity and the dynamics of vehicular interactions. Perhaps, no finite set of trust parameters can entirely represent the real-world behaviors of vehicles.
• Lack of an IoV-based trust testbed – Our efforts were primarily limited to the development of an IoV-based simulator, which, whilst useful, cannot fully replicate the intricacies of the real-world vehicular interactions. Therefore, the development of a real-world IoV-based trust testbed is indispensable in a bid to enhance the accuracy and the applicability of trust evaluation mechanisms.

Therefore, the envisaged trust-based IoV dataset not only provides researchers with the trust scores of the vehicles but also enables effective identification of the malicious vehicles and the respective attacks instigated by them through analysis of trust score fluctuations vis-à-vis time. As the first trust-based IoV dataset encompassing information pertinent to the trust-based attacks, it offers a strong support for future research in this particular domain.

## Ethics Statement

This study does not involve any human participants, animal experiments, or sensitive data. No ethical approval was, therefore, required.

## Credit Author Statement

Yingxun Wang: Conceptualization, Methodology, Data curation, Formal analysis, Software, Visualization, Writing – Original draft; Adnan Mahmood: Conceptualization, Methodology, Data curation, Formal analysis, Project administration, Validation, Supervision, Writing –Review and Editing; Xiang Wang: Conceptualization, Visualization, Funding acquisition.

## Data Availability

Trust – IoV: An Open Benchmark Dataset for Trust Management in the Internet of Vehicles (Original data)

## Appendix Supplementary materials

Image, application 1

## Footnotes

1 https://doi.org/10.5281/zenodo.17636200
